# The Prevalence of Dietary Supplement Use for the Purpose of COVID-19 Prevention in Japan

**DOI:** 10.3390/nu14153215

**Published:** 2022-08-05

**Authors:** Tsuyoshi Chiba, Nanae Tanemura

**Affiliations:** Department of Food Function and Labeling, National Institute of Health and Nutrition, National Institutes of Biomedical Innovation, Health and Nutrition, Tokyo 162-8363, Japan

**Keywords:** COVID-19, dietary supplements, information source

## Abstract

COVID-19 is still the biggest issue worldwide. Many dietary supplements on the market claim to have anti-COVID-19 effects without scientific evidence. To elucidate the prevalence of dietary supplement usage for the prevention of COVID-19, we conducted an online cross-sectional questionnaire survey among Japanese adults in January 2022. The prevalence of dietary supplement use for the prevention of COVID-19 was 8.3%, and there was no gender difference. We also conducted additional research on these dietary supplement users (1000 males and 1000 females). The most popular ingredient used was vitamin C (61.0%), with vitamin D (34.9%) and probiotics (33.4%) following. Half of these participants reported using supplements for more than one year. The information sources that reportedly led them to start using dietary supplements for the prevention of COVID-19 were the Internet (44.0%), television and radio (29.9%), and family or friends (26.0%), and these information sources differed among generations. In conclusion, some of the population used vitamin/mineral supplements for the prevention of COVID-19 that might be beneficial for their health, but some used ingredients with no scientifically proven effects against the virus at this time. Therefore, information-based scientific evidence is important to prevent the inappropriate use of dietary supplements by consumers.

## 1. Introduction

The novel coronavirus disease COVID-19 still influences lives around the world. Although several anti-SARS-CoV-2 vaccines and medicines have been developed [1,2], the cumulative number of cases and deaths are still increasing [3]. Some countries have conducted lockdowns to prevent community-acquired infection at the local level and have limited immigration to prevent the transportation of new/mutated viruses at the national level. However, many countries have moved to the next step to sustain economic activity, with a new lifestyle of living with COVID-19 having been proposed. In this regard, it is important to prevent SARS-CoV-2 infection as much as possible. Most national governments have encouraged their people to avoid the three Cs (closed spaces, crowded places, and close-contact settings) from the beginning of the pandemic. On the contrary, a lot of research into the cause of SARS-CoV-2 infection and COVID-19 severity has been conducted, and it has been reported that malnutrition, especially vitamin A, vitamin D, zinc, and selenium deficiencies, is associated with SARS-CoV-2 infection and COVID-19 severity [4,5].

The World Health Organization Regional Office for the Eastern Mediterranean (WHO EMRO) released “Nutrition advice for adults during the COVID-19 outbreak.” This advice includes the following recommendations: (1) Eat fresh and unprocessed foods every day, (2) drink enough water every day, (3) eat moderate amounts of fat and oil, (4) eat less salt and sugar, and (5) avoid eating out [6]. These recommendations are almost the same as the “Healthy Diet” recommendations published by the WHO before the pandemic [7]. This means that any special actions are not necessary under this pandemic if these usual healthy diet recommendations are followed. However, it is difficult to maintain a well-balanced diet in everyday life. Insufficiencies/deficiencies in some nutrients have been reported even in developed countries. In the USA, the National Health and Nutrition Examination Survey (2007–2010) showed that vitamin A, vitamin C, vitamin D, vitamin E, vitamin K, folate, magnesium, calcium, and potassium levels were lower than the Estimated Average Requirements (EAR) in almost all populations [8]. In Japan, deficiencies in vitamin A, vitamin B_1_, vitamin B_2_, and some minerals among Japanese female junior high school students have been reported, with skipping breakfast highlighted as a potential cause [9]. In addition, it has also been reported that people who frequently eat out have low intakes of vitamin C and some minerals [10].

Nutritional status is important for maintaining the immune system, with malnutrition attenuating the immune system [11]. Indeed, it has been reported that malnutrition is associated with an increased risk of SARS-CoV-2 infection, severity, and mortality [12]. Among nutrients, the influences of deficits of vitamin C, vitamin D, zinc, and selenium are well studied [13]. Today, some dietary supplements on the market that contain these vitamins and minerals claim anti-COVID-19 effects, even though there is no evidence that these dietary supplements can prevent COVID-19 in healthy people. Therefore, the WHO has informed consumers that “Micronutrients, such as vitamins D and C and zinc, are critical for a well-functioning immune system and play a vital role in promoting health and nutritional well-being. There is currently no guidance on the use of micronutrient supplements as a treatment of COVID-19.” [14].

Sex, age, house-income, and education level were associated with dietary supplement usage [15,16]. In addition, anxiety about health was also associated with dietary supplement use [16]. In this situation, some dietary supplements claim to prevent SARS-CoV-2 infection and severity of COVID-19 without scientific evidence not only in Japan, but also in the rest of world. At this time, it is illegal to claim prevention and treatment of disease on dietary supplements in Japan. The Consumer Affairs Agency in Japan has cautioned on these dietary supplements several times [17]. However, we reported that some Japanese consumers use fortified foods and dietary supplements not only to supplement their nutrient intake, but also to prevent infectious diseases [18]. Therefore, we conducted an online cross-sectional questionnaire survey to clarify the prevalence of the use of dietary supplements to prevent SARS-CoV-2 infection.

## 2. Materials and Methods

### 2.1. Participants and Procedures

An online cross-sectional survey was conducted by Neo Marketing Inc. (Tokyo, Japan) from 19 to 24 January 2022. The 48,925 respondents, aged 20–79 years old, comprised 25,739 males and 23,186 females that were randomly chosen from the research company’s monitors. This study was conducted with the approval of the Research Ethics Committee of the National Institutes of Biomedical Innovation, Health and Nutrition (No. 358, approved on 22 December 2021) and in accordance with the Declaration of Helsinki. Participants who had never been diagnosed COVID-19 and who were currently using dietary supplements for the prevention of COVID-19 were selected from the preliminary survey and were asked to answer a targeted survey questionnaire. In the targeted survey, questions were included about the dietary supplements that they used, their information sources, and whether they recognized the National Institutes of Biomedical Innovation, Health and Nutrition (NIBIOHN) COVID-19-related website (https://hfnet.nibiohn.go.jp/notes/detail.php?no=2142.html, Japanese only, accessed on 25 July 2022) (Supplementary File S1). Detailed procedures of the Internet survey are described in our previous report [18].

### 2.2. Statistical Analysis

Differences in the distribution among groups were compared using the chi-squared (*χ*^2^) test. All statistical analyses were performed using IBM SPSS Statistics ver. 28.0.1.0 (IBM Corporation, Armonk, NY, USA), and a *p*-value of <0.05 was considered statistically significant. Dietary supplement usage is influenced by consumers’ background (sex, age), and dietary supplement users tend to have a positive image about the prevention and treatment of diseases with dietary supplements [16]. In addition, a cumulative number of newly confirmed SARS-CoV-2 infection per 100,000 population in their resident prefecture [19] might have influenced to use dietary supplements. These factors were entered into bivariate logistic regression, and candidate variables were entered into the multivariable logistic regression model. To identify the factors of the prevalence of dietary supplement usage for the prevention of SARS-CoV-2 infection, binary logistic regression was done to assess. All variables having a *p*-value ≤ of 0.2 were considered as candidates for multivariable logistic regression and calculated odds ratio (OR) and 95% confidence interval (95% CI) using EZR ver. 4.0.3, which is a modified version of R commander designed to add statistical functions frequently used in biostatistics.

## 3. Results

### 3.1. Characteristics

The 48,925 respondents (46.9 ± 14.7 years old) comprised 25,739 males (20 s = 2960; 30 s = 3770; 40 s = 4433; 50 s = 8275; >60 s = 6301) and 23,186 females (20 s = 5630; 30 s = 4667; 40 s = 5028; 50 s = 4427; >60 s = 3704) (Table 1). Participants’ areas of residence were as follows: Hokkaido = 2566 (5.2%); Tohoku = 4336 (8.9%); Kanto = 17,229 (35.2%); Hokuriku = 1303 (2.7%); Tokai = 6205 (12.7%); Kinki = 7975 (16.3%); Chugoku/Shikoku = 4510 (9.2%); and Kyushu/Okinawa = 4801 (9.8%). This distribution was in accordance with the population distribution of Japan [20].

### 3.2. Experience of SARS-CoV-2 Infection

Data on participants’ experiences of SARS-CoV-2 infection are shown in Table 2. Only 1.9% of the participants had been diagnosed with COVID-19. However, 2.0% of the participants had experienced symptoms but had not visited a clinic. In the case of the people around them, 24.9% of them answered that some people around them had experienced SARS-CoV-2 infection, such as school friends or workplace colleagues (11.5%), acquaintances (8.9%), and family members (5.1%).

### 3.3. The Prevalence of Dietary Supplement Usage for the Prevention of SARS-CoV-2 Infection

The prevalence of dietary supplement usage for the prevention of SARS-CoV-2 infection is shown in Table 3. Most participants (91.7%) reported not currently using dietary supplement for the prevention of SARS-CoV-2 infection, but 8.3% reported that they did. There was no gender difference. The most common reason given for not using dietary supplements was “I don’t think that they can be prevented with dietary supplements” (57.6%), followed by “I think we have sufficient infection control measures (wearing masks, hand washing, gargling, avoiding 3C’s)” (40.9%) and “I can’t afford to buy them” (19.0%). Only 1.3% and 2.9% of the participants had seen the “Information for Consumers Related to COVID-19” published by the Consumer Affairs Agency and NIBIOHN. In addition, we analyzed the prevalence of dietary supplement usage for the prevention of SARS-CoV-2 infection according to experiences of SARS-CoV-2 infection (Table 4). It was highest for those participants who had been diagnosed with COVID-19 (37.9%). The next highest prevalence was for those participants whose family members (live-in) had been diagnosed with COVID-19 (29.3%), followed by those participants who had had symptoms (21.0%). For residence area, the prevalence was the highest for Kanto, which includes Tokyo (9.1%), and the lowest was for Tohoku (6.9%).

The prevalence of dietary supplement usage other than for the prevention of SARS-CoV-2 infection is shown in Table 5. The prevalence of this was higher than that for the prevention of SARS-CoV-2 infection. A total of 37.4% of participants answered “I currently use dietary supplements other than for the prevention of SARS-CoV-2 infection”, with females (40.1%) reporting a significantly higher prevalence than males (35.0%).

The results of the logistic regression analysis for the identification of factors influencing the dietary supplement usage for the prevention of SARS-CoV-2 infection are shown in Table 6. No multicollinearity was suggested by the correlation analysis among the independent variables. In this survey, sex, age, cumulative number of newly confirmed SARS-CoV-2 infection per 100,000 population of their residential prefecture, and the prevalence of dietary supplement usage other than for the prevention of SARS-CoV-2 infection were identified as significant factors.

### 3.4. Ingredients for the Prevention of SARS-CoV-2 Infection

Furthermore, we asked the 2000 participants who reported using dietary supplements for the prevention of SARS-CoV-2 infection which kinds of ingredients they used (Table 7). Most of them used vitamin/mineral supplements, such as vitamin C (61.0%), vitamin D (34.9%), vitamin E (31.2%), zinc (23.8%), and iron (21.1%). Some of them used non-vitamin/non-mineral supplements, with the most popular ingredients being probiotics (33.4%). In this survey, 2.0% of participants used cannabidiol (CBD) for the prevention of SARS-CoV-2 infection. Most participants used 1 product (37.8%), followed by 2 products (22.4%), 3 products (17.2%), 4 products (9.5%), 5 products (6.2%), and 6 products or more (7.1%). The most popular combination was vitamin C and vitamin D (25.1%), then vitamin C and vitamin E (24.6%), vitamin D and vitamin E (19.6%), and vitamin C and probiotics (18.9%) were following. In addition, “others” included 5-aminolaevulinic acid, DHA/EPA, garlic, propolis, and so on.

### 3.5. Duration of Dietary Supplement Use for the Prevention of SARS-CoV-2 Infection

Data on the duration of dietary supplement use for the prevention of SARS-CoV-2 infection are shown in Table 8. If participants used more than one dietary supplement, they answered the duration of longest one. In total, almost half of the participants reported using dietary supplements for more than one year (53.1%), with 12.8% reporting using them for four to six months, and 12.6% for two to three months (12.6%). Some participants had only started them one week prior (5.4%), because we conducted the survey at a point when the number of SARS-CoV-2 PCR-positive patients was increasing in Japan.

### 3.6. Information Source That Prompted Participants to Use Dietary Supplements for the Prevention of SARS-CoV-2 Infection

Data on the information source that prompted the participants to use dietary supplements for the prevention of SARS-CoV-2 infection are shown in Table 9. Among all participants, the Internet was the highest reported source (44.0%), followed by television or radio (29.9%), family or friends (26.0%), and SNS including LINE, Facebook, Twitter, Instagram (21.7%). It is well known that the information sources used differ among generations, so we analyzed these data according to generation. The Internet was the highest reported source for all generations except for participants in their twenties. For these, SNS was the highest (35.3%), which was the lowest for those in their seventies. Surprisingly, specialists were a higher reported source for young generations than for old generations.

### 3.7. The Perception and Usefulness of “Information for Consumers Related to COVID-19” Published by the NIBIOHN

Data on the perception and usefulness of “Information for Consumers Related to COVID-19” published by the NIBIOHN are shown in Table 10. For those participants who reported using dietary supplements for the prevention of SARS-CoV-2 infection, 15.3% of them knew and 17.6% had seen this site. Among them (657 participants), half of them answered that “This site is useful to some extent” (51.1%), with 28.9% answering that “This site is very useful” (Table 11). On the contrary, 67.2% of them answered that “I have never seen this site” (Table 10). However, most of them answered that “This site seems useful to some extent” (60.5%) and “This site seems very useful” (15.7%) (Table 12).

Finally, we asked participants whether they would stop using the dietary supplements that they used for the prevention of SARS-CoV-2 infection or not; only 3.3% of them answered “I would stop it immediately.” and 18.2% of them answered “I would stop it after it run out.”

## 4. Discussion

Due to the COVID-19 pandemic, some people have been using dietary supplements for the prevention of SARS-CoV-2 infection. In the present study, it was found that the prevalence of dietary supplement use was higher for those participants who had experienced SARS-CoV-2 infection than for others. Multivariable regression analysis showed that sex, age, cumulative number of newly confirmed SARS-CoV-2 infection per 100,000 population of their residential area, and other dietary supplement usage, were identified as significant factors of dietary supplement usage for the prevention of SARS-CoV-2 infection. Among these factors, other dietary supplement usage was the strongest factor to use dietary supplement for the prevention of SARS-CoV-2 infection. Most participants reported obtaining information about COVID-19 from the Internet. The NIBIOHN provides evidence levels of dietary supplement ingredients for upper respiratory infections, including COVID-19, via their Internet website, but almost 70% of the participants reported not knowing about this site.

Two years have passed since COVID-19 was declared a pandemic by the WHO on 11 March 2020. Currently, vaccination is being carried out continuously, and therapeutic drugs have been put into practical use, but the momentum of infection has not yet subsided, and the number of infected people is increasing repeatedly [3]. Therefore, the prevention of SARS-CoV-2 infection is of the utmost importance. In these circumstances, since information on dietary supplements and ingredients that claim to be good for preventing new coronavirus infections, but which lack scientific evidence, is available on the Internet [21,22], the NIBIOHN have launched a new coronavirus information site to provide consumers with scientific evidence-based information (Japanese only). This site has been introduced several times in the media, and it is also displayed as the top-ranking result when the keywords “corona virus” and “health food” are searched for on Google Japan. Therefore, in this study, we investigated the awareness of this site among Japanese people, and found that 33% of those participants who were dietary supplement users for the purpose of preventing SARS-CoV-2 infection were aware of the site, and most of them were satisfied with it. This result indicates that this website might be helpful for consumers when choosing dietary supplements.

In this survey, there were generational differences in information sources that prompted the participants to use dietary supplements for the prevention of SARS-CoV-2 infection. SNS was more popular in younger generations. It might be the cause of the higher prevalence of dietary supplement use for the prevention SARS-CoV-2 infection in younger generations, because SNS can be a platform for fake news [23]. On the other hand, specialists were also a higher reported source for younger generations than for older generations. There are some speculations about this situation. First, health literacy might be higher in younger generations than in older generations [24,25]. We also reported that awareness of health support pharmacies was higher in younger generations than in older generations [26]. Health support pharmacies is a new category of pharmacies in Japan, and not only patients but also community residents can consult with professionals about any aspect, including dietary supplement usage. Second, old age is one of the risk factors for severity and mortality of COVID-19 [27]. So, the older generations might avoid going out and contacting other people. However, most of them take medications and should consult with health care professionals about dietary supplement use.

To prevent COVID-19, a well-balanced diet is encouraged [6,28]. However, our previous study indicated that only one-third of Japanese people consumed a well-balanced diet almost every day during the COVID-19 pandemic [18]. In this regard, some Japanese people might have some nutrient deficiencies. Deficiencies/insufficiencies in vitamin D [29,30,31], vitamin C [32], zinc [29], magnesium [29,33], and selenium [34] have been associated with COVID-19 infection and severity. In addition, supplementing COVID-19 patients’ vitamin D intake has been found to decrease ICU admission rates [35]. On the contrary, vitamin C [36] and zinc [37] supplementation for COVID-19 patients has failed to show any observable benefits. At this time, there is no evidence that dietary supplements that contain these nutrients can prevent SARS-CoV-2 infection, but insufficiencies/deficiencies in these nutrients are associated with risk of SARS-CoV-2 infection, severity, and mortality [12]. In addition, a well-balanced diet is also associated with a lower risk of mortality [38,39,40,41]. In this survey, we did not ask the participants about their dietary habits, so we did not define the association between dietary habits and COVID-19 diagnosis. However, we recommend a well-balanced diet or the appropriate use of vitamin/mineral supplements to maintain one’s nutritional condition and reminded consumers not to overdose. Excess intake of each vitamin/mineral is also associated with adverse effects, so dietary reference intakes for Japanese people sets a tolerable upper intake level (UL) of each vitamin/mineral [42], as well as other authorities [43,44].

It is reported that a lot of people used dietary supplements and over-the counter drugs as self-medication for the prevention and treatment of COVID-19, and a systematic review was conducted [45]. This systematic review included 14 studies from 14 countries (Japan was not included), and the prevalence of self-medication for the prevention of COVID-19 was 44.9% (from 3.9% to 96.2% among studies). In this report, the prevalence of vitamins (vitamin B, C, D, E, folic acid, and multivitamins) was 64%, herbal and natural products (ginger, honey, propolis, garlic, ginkgo biloba, omega-3 fatty acids) was 50%, minerals (zinc, calcium, iron, magnesium) was 43%, and most ingredients corresponded with our results. In addition, it is reported that some herbal supplement sales increased in the USA during COVID-19 pandemic [46]. Sales of elderberry, ashwagandha, apple cider vinegar, ginger, and echinacea were increased, but some of them were not reported in our survey. This might be due to the different regulations concerning herbal products between Japan and USA. On the other hand, CBD sales decreased in the USA during the COVID-19 pandemic [46].

In this study, 2.0% of those participants who reported using dietary supplements for the prevention of SARS-CoV-2 infection reported using CBD. CBD has become popular recently not only in Japan, but across the world. CBD is one of the cannabinoids derived from hemp (*Cannabis sativa*). Different from delta-9-tetrahydrocannabinol (THC), CBD is known as a non-psychoactive cannabinoid and seems safe under appropriate usage. CBD shows anti-inflammatory properties [47] and inhibitory effects on SARS-CoV-2 infection [48] and replication [49]. However, only two reports have been conducted on humans. One of the studies reported that CBD administration in COVID-19 patients failed to prevent the deterioration of the clinical status of COVID-19 symptoms [50], whereas the other study reported that CBD administration in healthcare workers during the COVID-19 pandemic could reduce the symptoms of burnout and emotional exhaustion [51]. This means that there is no evidence that CBD can prevent or reduce SARS-CoV-2 infection or severity in human studies, but it might be effective for reducing pandemic-related stress. However, it has been reported that excess CBD intake can cause adverse effects, such as hepatitis [52], respiratory depression [53], and diarrhea [54]. In addition, interactions with drugs have also been reported [55,56], so patients who take medication should not use CBD based on their own judgment.

This study was conducted through an online survey, which is both a strength and a limitation. Due to the pandemic, face-to-face and mail surveys are difficult to carry out. However, an online survey offers easy and contact-free participation. In addition, many people receive information about health, dietary supplements, drugs, and diseases via the Internet [57]. This means that the registrants of online survey companies might correspond to the target population of dietary supplement users. However, many consumers also receive information from mass media other than the Internet, which means that our study could not gather data on people who do not use the Internet and belong especially to older generations. In addition, this survey was conducted in January 2022, but anxiety among consumers might have been highest during the stay-at-home request in Japan (March–April to May 2020), and this might be associated with starting dietary supplement use for COVID-19 prevention. In addition, we found a higher prevalence of dietary supplement usage in participants who had experienced SARS-CoV-2 infection. However, we did not ask whether they had begun using supplements before or after they were diagnosed, and whether they had taken adequate infection control measures. In today’s circumstances, it is not clear whether dietary supplements are helpful for COVID-19 prevention, although vitamin/mineral supplements might help maintain people’s nutritional condition. In this study, some participants reported using dietary supplements that have no scientifically proven effects against COVID-19, such as catechins, echinacea, and CBD. To avoid the inappropriate use of dietary supplements, consumers should be provided with relevant information.

## 5. Conclusions

In this study, 8.3% of the participants reported using dietary supplements for the prevention of SARS-CoV-2 infection, and they mainly used nutrients, such as vitamin C, vitamin D, and zinc. These nutrients might be beneficial for their health by helping to avoid excess nutritional intake, even though there is no scientific evidence that they can help prevent SARS-CoV-2 infection. However, some participants reported using non-nutrient ingredients, such as probiotics, catechin, and CBD. CBD is generally recognized as safe, but there is no evidence that CBD is effective for the prevention of SARS-CoV-2 infection, and adverse events have also been reported. Therefore, the inappropriate use of these dietary supplements should be avoided. In addition, dietary supplement usage other than for the prevention of SARS-CoV-2 infection was a strong factor of dietary supplement usage for the prevention of SARS-CoV-2 infection. It is important to provide consumers, especially current supplement users, with information for proper use.

## Figures and Tables

**Table 1:** Characteristics of respondents.

	*n*	%
Number	48,925	
Sex		
Male	25,739	52.6
Female	23,186	47.4
Age		
20 s	8320	17.0
30 s	8437	17.2
40 s	9461	19.3
50 s	12,702	26.0
Over 60 s	10,005	20.4
Areas of residence		
Hokkaido	2566	5.2
Tohoku	4336	8.9
Kanto	17,229	35.2
Hokuriku	1303	2.7
Tokai	6205	12.7
Kinki	7975	16.3
Chugoku/Shikoku	4510	9.2
Kyushu/Okinawa	4801	9.8

**Table 2:** Experience of SARS-CoV-2 infection.

	*n*	%
I have been diagnosed with COVID-19	775	1.9
I have not been diagnosed with COVID-19	4345	10.8
I have had symptoms, but did not visit a clinic	822	2.0
I have had no COVID-19 symptoms to date	34,436	85.3
People around ^1^		
Family members (live-in)	928	2.3
Family members (estranged)	1133	2.8
School friends or workplace colleagues	4641	11.5
Acquaintances	3593	8.9
Others	1237	3.1
No one	30,323	75.1

^1^ Multiple answers.

**Table 3:** The prevalence of dietary supplement usage for the prevention of SARS-CoV-2 infection.

	*n*	%	Male	Female	*p*-Value
I currently use dietary supplements for the prevention of SARS-CoV-2 infection	3362	8.3	8.4	8.2	0.541
I do not use dietary supplements for the prevention of SARS-CoV-2 infection	37,016	91.7	91.6	91.8	
Reasons for not using (*n* = 37,016) ^1^					
I am not worried about SARS-CoV-2 infection	4196	11.3	14.4	7.8	<0.001
I take adequate infection control measures (wearing masks, hand washing, gargling, avoiding 3C’s)	15,137	40.9	41.9	39.8	<0.001
I do not think that dietary supplements can prevent coronavirus infection	21,328	57.6	55.8	59.7	<0.001
I cannot afford to buy them	7037	19.0	17.9	20.3	<0.001
I have seen “Information for Consumers Related to COVID-19” by the Consumer Affairs Agency	486	1.3	1.5	1.1	0.006
I have seen “Information for Consumers Related to COVID-19” by the NIBIOHN	1091	2.9	3.1	2.8	0.106
Others	4196	11.3	14.4	7.8	<0.001

CAA, Consumer Affairs Agency in Japan; NIBIOHN, National Institutes of Biomedical Innovation, Health and Nutrition. The difference among groups was examined using the chi-square (*χ*^2^) test. ^1^ Multiple answers.

**Table 4:** The prevalence of dietary supplement usage for the prevention of SARS-CoV-2 infection according to the experience of SARS-CoV-2 infection.

	*n*	%
I have been diagnosed with COVID-19	294	37.9
I have not been diagnosed with COVID-19	617	14.2
I have had symptoms, but did not visit a clinic	173	21.0
I have never had symptoms	2273	6.6
People around ^1^		
Family members (live-in)	272	29.3
Family members (estranged)	205	18.1
School friends or workplace colleagues	552	11.9
Acquaintances	481	13.4
Others	108	8.7
No one	2062	6.8

^1^ Multiple answers. Each % was calculated as *n* divided by each *n* in Table 2.

**Table 5:** The prevalence of dietary supplement usage other than for the prevention of SARS-CoV-2 infection.

	*n*	%	Male	Female	*p*-Value
I currently use dietary supplement other than for the prevention of SARS-CoV-2 infection	15,097	37.4	35.0	40.1	<0.001
I do not use dietary supplements other than for the prevention of SARS-CoV-2 infection	25,281	62.6	65.0	59.9	

The difference among groups was examined using the chi-square (*χ*^2^) test.

**Table 6:** Factors associated with dietary supplement usage for the prevention of SARS-CoV-2 infection.

	Univariable OR (95% CI)	*p*-Value	Multivariable OR (95% CI)	*p*-Value
Sex				
Male	reference		reference	
Female	0.978 (0.911–1.050)	0.541	0.733 (0.678–0.792)	<0.001
Age				
20 s	reference		reference	
30 s	1.000 (0.893–1.120)	0.993	0.881 (0.779–0.998)	0.005
40 s	0.887 (0.792–0.993)	0.037	0.719 (0.636–0.812)	<0.001
50 s	0.684 (0.612–0.763)	<0.001	0.493 (0.437–0.557)	<0.001
Over 60 s	0.713 (0.636–0.799)	<0.001	0.444 (0.392–0.503)	<0.001
Cumulative number of newly confirmed SARS-CoV-2 infection per 100,000 population of residential prefecture ^1^				
<15,000	reference		reference	
15,001–30,000	1.100 (0.998–1.220)	0.055	1.150 (1.030–1.270)	0.001
30,000< ^2^	1.230 (1.120–1.340)	<0.001	1.200 (1.090–1.320)	<0.001
Dietary supplement usage other than for the prevention of SARS-CoV-2 infection				
No	reference		reference	
Yes	20.60 (18.30–23.20)	<0.001	22.30 (19.80–25.10)	<0.001

^1^ Cumulative number from 16 Jun 2020 to 18 Jun 2022 (the day before the questionnaire). ^2^ Hyogo, Saitama, Chiba, Kanagawa, Osaka, Tokyo, and Okinawa in this segment.

**Table 7:** Dietary supplements that the participants reported using for the prevention of SARS-CoV-2 infection.

	*n*	%
Vitamin/Mineral		
Vitamin C	1199	61.0
Vitamin D	686	34.9
Vitamin E	614	31.2
Zinc	467	23.8
Iron	415	21.1
Non-vitamin/non-mineral		
Probiotics	657	33.4
Catechins	367	18.7
Echinacea	56	2.8
Cannabidiol (CBD)	40	2.0
Others	253	12.9

Multiple answers.

**Table 8:** Duration of dietary supplement use.

	*n*	%
Within 1 week	108	5.4
1 month	198	9.9
2–3 months	251	12.6
4–6 months	255	12.8
7–11 months	126	6.3
More than 1 year	1062	53.1

*n* = 2000.

**Table 9:** Information sources that prompted the participants to use dietary supplements for the prevention of SARS-CoV-2 infection.

	All	20 s	30 s	40 s	50 s	60 s	70 s	*p*-Value
Television or radio	29.9	24.8	31.3	30.0	29.0	34.1	34.5	0.102
Newspaper, magazine, or advertisement	16.1	17.5	15.0	14.3	13.5	16.1	28.1	0.002
Internet	44.0	35.0	47.0	47.0	46.0	48.7	38.1	<0.001
SNS (LINE, Facebook, Twitter, Instagram)	21.7	35.3	29.0	21.3	16.8	7.7	2.9	<0.001
Specialists (doctors, pharmacists, dieticians)	9.6	13.5	12.3	6.0	9.0	7.3	7.2	0.002
Store clerks in pharmacies or drugstores	12.0	13.0	15.8	11.3	11.0	9.6	7.9	0.077
Point-of-purchase adverts	9.4	8.3	11.8	11.0	9.0	9.2	2.9	0.043
Product packaging	15.2	10.8	17.3	19.0	18.5	10.3	10.8	<0.001
Family, friends, or acquaintances	26.0	25.0	27.0	22.3	27.5	26.8	30.2	0.394
Others	3.5	1.8	3.3	3.8	4.0	3.4	7.2	0.085

Multiple answers. The difference among minerals was examined using the chi-square (*χ*^2^) test.

**Table 10:** The perception of “Information for Consumers Related to COVID-19” published by the NIBIOHN.

	*n*	%
I know this site	306	15.3
I have seen this site	351	17.6
I have never seen this site	1343	67.2

*n* = 2000.

**Table 11:** The usefulness of “Information for Consumers Related to COVID-19” published by the NIBIOHN for users.

	*n*	%
This site is very useful	190	28.9
This site is useful to some extent	336	51.1
This site is not very useful	103	15.7
This site is not useful at all	23	3.5
I know this site, but I have not seen the information on it	5	0.8

*n* = 657 who answered “I know this site” and “I have seen this site” in Table 10.

**Table 12:** The usefulness of “Information for Consumers Related to COVID-19” published by the NIBIOHN for non-users.

	*n*	%
This site seems very useful	211	15.7
This site seems useful to some extent	812	60.5
This site seems not very useful	241	17.9
This site seems totally useless	79	5.9

*n* = 1343 who answered “I have never seen this site” in Table 10.

## Data Availability

The data presented in this study are available upon request from the corresponding author.

## Supplementary Materials

The following supporting information can be downloaded at: https://www.mdpi.com/article/10.3390/nu14153215/s1. File S1: Questionnaire.
