# Supplementary material for: The Prevalence of Dietary Supplement Use for the Purpose of COVID-19 Prevention in Japan
Source: Nutrients. 2022 Aug 5;14(15):3215. doi: 10.3390/nu14153215 (PMC9370294; doi:10.3390/nu14153215)
Supplement: Supplementary file 1 [file nutrients-14-03215-s001.zip › nutrients-1828549-supplementary.pdf]

Questionnaire

Preliminary survey

SC1 Please inform about your situation with new coronavirus infection.

1. I have been diagnosed with COVID-19.
2. I have not been diagnosed with COVID-19.
3. I have had symptoms, but did not visit a clinic.
4. I have no COVID-19 symptoms to date.

SC2 Has anyone close to you been infected with a new type of coronavirus?

1. No one close to you has been infected
2. Family members (live-in)
3. Family members (estranged)
4. School friends or workplace colleagues
5. Acquaintances
6. Others

SC3 Do you currently use dietary supplements to prevent new coronavirus infection?

1. Yes, I currently use dietary supplements for the prevention of SARS-CoV-2 infection.
2. No, I do not use dietary supplements for the prevention of SARS-CoV-2 infection.

This question for those who answered 'No' on SC3.

SC4 What are reasons for not using dietary supplements to prevent new coronavirus infection?

1. I am not worried about SARS-CoV-2 infection.
2. I take adequate infection control measures (wearing masks, hand washing, gargling, avoiding 3C's).
3. I do not think that dietary supplements can prevent coronavirus infection.
4. I cannot afford to buy them.
5. I have seen "Information for Consumers Related to COVID-19" by the Consumer Affairs Agency.
6. I have seen "Information for Consumers Related to COVID-19" by the National Institutes of Biomedical Innovation, Health and Nutrition.
7. Others

SC5 Do you currently use dietary supplements for purposes other than prevention of SARS-CoV-2 infection?

1. Yes, I do.
2. No, I do not.

Actual survey

Q1 Which dietary supplements do you use to prevent new coronavirus infection?

1. Vitamin C
2. Vitamin D
3. Vitamin E
4. Zinc
5. Iron
6. Probiotics (e.g., lactic acid bacteria)
7. Catechins
8. Echinacea.
9. Cannabidiol (CBD).
10. Others

Q2 How long have you been using dietary supplements?
If you use more than one product, please indicate the one you have used the longest.

1. Within one week
2. 1 month
3. 2-3 months
4. 4-6 months
5. 7-11 months
6. More than 1 year

Q3 What prompted you to use the product?

1. Television or radio (including commercials)
2. Newspapers, magazines, or advertisements
3. Internet (product websites, word-of-mouth websites, summary websites)
4. Social Network Service (LINE, Facebook, Twitter, Instagram)
5. Specialists (doctors, pharmacists, dietitians)
6. Store clerks in pharmacies or drugstores.
7. Point-of-purchase adverts
8. Product packaging
9. Family, friends, or acquaintances.
10. Other

Q4 Have you seen this website?
(View https://hfnet.nibiohn.go.jp/notes/detail.php?no=2142: HFNet website)

1. Yes, I know this site.

2. Yes, I have seen this site.
3. No, I have never seen this site.

This question for those who answered 'Yes' on Q4.

Q4-2 Did you find the information on this website useful?

1. This site is very useful.
2. This site is useful to some extent.
3. This site is not very useful.
4. This site is not useful at all.
5. I know this site, but I have not seen the information on it.

This question for those who answered 'No' on Q4.

Q4-3 Is the information on this website likely to be useful for your future product choice?

1. This site seems very useful.
2. This site seems useful to some extent.
3. This site seems not very useful.
4. This site seems totally useless.

Q5 Will you stop using dietary supplements that you were using to prevent new coronavirus infection?

1. Yes, I would stop it immediately.
2. Yes, but I would stop it after it run out.
3. No, I would not stop it.

Thank you for your cooperation
